# Analyzing the Effects of Pretreatment Diversity on HCV Drug Treatment Responsiveness Using Bayesian Partition methods

**Published:** 2015-05-12

**Authors:** Yao Fu, Gang Chen, Xuan Guo, Jing Zhang, Yi Pan

**Affiliations:** 1 Department of Mathematics and Statistics, Georgia State University; 2 Program of Computational Biology and Bioinformatics, Yale University; 3 Department of Statistics, Yale University; 4 Department of Computer Science, Georgia State University, Atlanta, Georgia, USA

## Abstract

Traditional therapies for Hepatitis C Virus (HCV) often yield unsatisfactory results. The reason for this may lie in the mechanism of drug resistance of the HCV virus. Despite doing a plain vanilla comparison between the treated and untreated groups, this paper takes a detour and investigates the drug resistance mechanism to interferon plus ribavirin combined therapy by comparing pretreatment sequence data between response and non-response patients in the NS5A region for genotype 1a HCV virus. We use Bayesian probabilistic models to detect single mutation or mutation combinations, and infer interaction structures between these mutations, to investigate the drug resistance combinations differences between those patients. We hope to decipher, at least partially, the reason behind the unsatisfactory results received from interferon plus ribavirin therapy.

**Author Summary:**

HCV treatment results have been historically suboptimal^[1–3]^. HCV drug resistance, which further hinders the treatment effects, is caused by mutations of viral proteins that disrupt the drugs’ binding but do not affect the viral survival. Due to the high rate and low fidelity of HCV replication, resistant strains quickly become dominant in a viral population under the selection pressure of a drug. M.J. Donlin et al indicate that pretreatment sequence diversity correlates with response effects^[15]^. We incorporate this idea and use a Bayesian approach to look into the pretreatment sequences diversity of HCV virus between response and non-response groups, under a combined treatment of interferon and ribavirin.

## Introduction

As a single-strand RNA virus, Hepatitis C virus (HCV) has been categorized into at least six genotypes each with several subtypes. Spreading in different regions, the genotypes will have dissimilar response patterns to interferon-based therapy. In clinic, less than 20% of chronic patients show sustained response with interferon monotherapy treatment^[1]^, while the therapy with the IFN and ribavirin combination showed significant improvement in response rates^[2,3]^. An accurate interpretation of the mechanism behind the antiviral resistance to IFN therapy will be a key factor for developing better treatment strategies.

Many studies have found that variations in HCV sequences play a role in response to IFN-based therapies, especially for the variations in the NS5A region^[4,5]^. NS5A is a nonstructural protein which leads to the resistance of IFN treatment by blocking the function of an important mediator in IFN response, dsRNA dependent protein kinase (PKR)^[6,7]^. In 1995, an “interferon sensitivity determining region” (ISDR amino acid [aa] 2209–2248) defined by Enomoto et al. in NS5A, is enriched with mutations related to resistance to IFN^[8]^. This finding has been confirmed by several studies^[9–11]^. Moreover, some other study showed evidence that in PKR binding domain (PKRbd aa 2209–274) of NS5A mutations can hamper the viral replication^[12]^. However, conflicting results about these two regions are also found, which complicate the role of NS5A in response to IFN. In addition to ISDR and PKRbd, other domains in NS5A have also been concerned in resistance to virus, such as proline-rich region (PRR aa 2283–2327), AR1 (aa 2144–2185) and AR2 (aa 2221–2272) of nuclear localization signal (NLS), and the variable region 3 (V3 aa 2356–2379) in the C terminus^[13]^.

This study will concentrate on NS5A region particular for HCV genotype 1a. In NS5A region there are 1344 base pairs, linking to 448 amino acids. The goal of this paper is to compare the pretreatment sequence patterns between those patients who respond positively to the treatment and those who don’t, and then infer the possible mutation positions that may affect the treatment effects.

To better facilitate the discussion of our findings, we first introduce the inner structure of NS5A region. It constitutes the following regions: the membrane attachment region (aa 1–236); the carboxyl region (aa 237–448); and the regions within the carboxyl end, such as PKRbd (aa 237–302), variable region 4 (V4; aa 310–330), variable region 3 (V3; aa 381–409), the region between V4 and V3 (aa 331–380), and the downstream region of V3 (aa 410–448).

In this study, we employed Bayesian models, which are originally proposed by Zhang et al. for investigating mutation interactions of HIV caused by a certain drug treatment^[14]^. Based on the Bayesian variable partition (BVP) model, we first used Metropolis-Hastings algorithm on the data of the interferon treatment to sort out mutations associated with drug resistance, and then applied a recursive model selection (RMS) procedure on the selected mutation positions to infer the dependence structure with the interacting effects.

## Methods

Here we first employed the Bayesian variable partition (BVP) model^[15]^ to search for the mutation positions. After detecting the interaction mutation positions, we further applied the Recursive Model Selection^[16]^ (RMS) on selected mutations by BVP to infer more detailed dependence structure among the interacting positions.

### Bayesian Variable Partition Model

The response group and non-response group can be represented as two data matrices *A*=*[A_1_*,…,*A_m_]* (of dimension *N_A_*×*m*) and *B*=*[B_1_*,…,*B_m_]* (of dimension *N_B_*×*m*), respectively (each row is a sequence, each column is a position of protein HCV RT). Here *N_A_* or *N_A_* denotes the number of sequences in response or non-response group respectively, and m denotes the number of positions. The m positions can be partitioned into four sets: set 1 contains positions independent with each other sharing the same distribution in response and non-response groups; set 2 contains positions independent with each other but with different distributions in two groups; set 3 contains positions dependent with the same distribution in two groups; set 4 contains positions dependent with different distributions in two groups. These four sets are corresponding to the four hypotheses in the result section. Let *I*=*(I_1_*,…,*I_m_)* indicate the membership of the positions with *I_j_*=1,2,3 and 4, respectively, and *A*^(1)^ and *B*^(1)^ denote the sequences in *l^th^* set from two groups. Our goal is to infer the sets of positions with different distributions in two groups (that is *I_j_* = 2 or 4).

Assume that there are *c_j_* possible values (amino acid types) at position j, and let Θ_*1*_={(θ_*j1*_,θ_*j2*_,…,θ_*j_cj_*_):*I_j_*=*1*} be the amino acid frequencies of each position in set 1 in both groups, thus, the likelihood of (*A*^(1)^, *B*^(1)^) is

(1) $$P(A^{\left(1\right)},B^{\left(1\right)}|\Theta_{1})=\Pi_{j:I=1}\Pi_{k=1}^{c_{j}}\theta_{jk}^{n_{jk}}$$

where {n_j1_,…,n_*j_cj_*_} are number of sequences taking k^th^ value in (*A*^(1)^, *B*^(1)^). Assume a Dirichlet prior on Θ_1_, that is, Θ_1_~Dirichlet(α) where α(α_1_,…, α_*c_j_*_) By integrating out Θ_1_, we have the marginal probability:

(2) $$P(A^{\left(1\right)},B^{\left(1\right)}|I)=\Pi_{j:I=1}(\Pi_{k=1}^{c_{j}}\frac{\Gamma (n_{jk}+\alpha_{k})}{\Gamma (\alpha_{k})})\frac{\Gamma (|\alpha |)}{\Gamma (N+|\alpha |)}$$

where |α| is the sum of all elements in α, and *N*=*N_A_*+*N_B_*.

Different to positions in set 1, two priors, Dirichlet (β^A^) and Dirichlet (β^B^), are used on the amino acid frequencies of each positions in set 2 in group A and B, respectively. By integrating out frequencies, we obtain

(3) $$P(A^{\left(2\right)}|I)=\Pi_{j:1=2}(\Pi_{k=1}^{c_{j}}\frac{\Gamma (n_{jk}+\beta_{k}^{A})}{\Gamma (\beta_{k}^{A})})\frac{\Gamma (|\beta^{A}|)}{\Gamma (N_{A}+|\beta^{A}|)}$$

(4) $$P(B^{\left(2\right)}|I)=\Pi_{j:1=2}(\Pi_{k=1}^{c_{j}}\frac{\Gamma (n_{jk}+\beta_{k}^{B})}{\Gamma (\beta_{k}^{B})})\frac{\Gamma (|\beta^{B}|)}{\Gamma (N_{B}+|\beta^{B}|)}$$

Positions in set 3 and 4 influence the resistance statuses through interactions. Thus, each amino acid combination over set 3 or 4 represents a potential mutation. Assume there are c^(3)^ and c^(4)^ possible value combinations for set 3 and 4, respectively, we use *Dirichlet*(γ) prior on the combination frequencies in set 3 and use two priors, *Dirichlet* (δ^A^) and *Dirichlet*(δ^B^), on the combination frequencies in set 4 for response group and non-response group. By integrating out frequencies, we obtain

(5) $$P(A^{\left(3\right)},B^{\left(3\right)}|I)=\Pi_{j:I=3}(\Pi_{k=1}^{c^{\left(3\right)}}\frac{\Gamma (n_{jk}+\gamma_{k})}{\Gamma (\gamma_{k})})\frac{\Gamma (|\gamma |)}{\Gamma (N+|\gamma |)}$$

(6) $$P(A^{\left(4\right)}|I)=\Pi_{j:I=4}(\Pi_{k=1}^{c^{\left(4\right)}}\frac{\Gamma (n_{jk}+\delta_{k}^{A})}{\Gamma (\delta_{k}^{A})})\frac{\Gamma (|\delta^{A}|)}{\Gamma (N_{A}+|\delta^{A}|)}$$

(7) $$P(B^{\left(4\right)}|I)=\Pi_{j:I=4}(\Pi_{k=1}^{c^{\left(4\right)}}\frac{\Gamma (n_{jk}+\delta_{k}^{B})}{\Gamma (\delta_{k}^{B})})\frac{\Gamma (|\delta^{B}|)}{\Gamma (N_{B}+|\delta^{B}|)}$$

Combining formulas from (1) to (7), we have the posterior distribution of I as

(8) $$P(I|A,B)aP(A^{\left(1\right)},B^{\left(1\right)}|I)P(A^{\left(2\right)}|I)P(B^{\left(2\right)}|I)P(A^{\left(3\right)},B^{\left(3\right)}|I)P(A^{\left(4\right)}|I)P(B^{\left(4\right)}|I)P(I)$$

In this study, we assume most positions should be in set 1 or set 3 in prior (i.e. unassociated with drug resistance), P(I_i_=2)=P(I_i_=4)=0.01, and $P(I)=\Pi_{i=1}^{m}P(I_{i})$. We further set the parameters for all Dirichlet priors to 0.5. A Markov chain Monte Carlo (MCMC) algorithm^[17]^ can be designed to sample from this posterior distribution so as to infer which variables are associated with the treatment status. More details on BVP can be found in^[18]^.

### Recursive Model Selection

We applied the RMS procedure to infer the detailed dependence structure among the interacting positions generated by BVP. Our strategy is to recursively apply a model selection of two classes of cruder models, that is the chain-dependence model and the V-dependence model, until the data do not support more detailed models.

We say that a group of variables X_G_ follow a chain-dependence model if the index set G can be partitioned into three subsets U, V, and W such that X_U_ and X_W_ are independent given X_V_, such as X_U_→X_V_→X_W_. The joint distribution of a chain-dependence model is

(9) $$P(X_{G})=P(X_{U})P(X_{V}|X_{U})P(X_{W}|X_{V})$$

We say that a group of variables X_G_ follow a V-dependence model if X_U_ and X_W_ are mutually independent, that is X_U_→X_W_←X_V_. The joint distribution of a V-dependence model is

(10) $$P(X_{G})=P(X_{U})P(X_{W})P(X_{V}|X_{U},X_{W})$$

In these two models, only set W is allowed to be empty, in which case these models become the saturated model.

We use a model indicator $I^{CV}=(I_{1}^{CV},I_{2}^{CV},\ldots ,I_{L}^{CV})$ to imply the membership of the L positions with $I_{j}^{cv}=0$ for the chain-dependence model and $I_{j}^{cv}=1$ for the V-dependence model. Let 𝕻 denote the set partition, the posterior distribution of I^CV^ and 𝕻 is

(11) $$P(𝔓,I^{CV}|\text{Data})\propto P(\text{Data}|𝔓,I^{CV})P(𝔓)P(I^{CV})$$

We set equal prior probability for I^CV^ and 𝕻 is An MCMC algorithm is designed to simulate from (11) and to find the optimal model type and variable partition. More details on RMS can be found in^[13]^. The procedure is applied recursively until only single-variable nodes are available.

BVP model and RMS procedure were utilized sequentially to the data of response and non-response patients. For the comparison, we applied BVP to 47 response datasets versus 29 non-response dataset, and contrived the difference between these two, recognizing that there exist different pretreatment patterns that we should account for differently. The detailed accession numbers for total 76 sequences are showed in Table 1, 2. More information about these sequences can be accessed in the reference^[19–20]^.

## Results

As M.J. Donlin et al. indicated, pretreatment sequence diversity correlates with response effects^[15]^. On observing this finding, our analysis is to test the following four proposed hypotheses. Hypothesis 1: the positions are independent with each other, and the probability distribution of the pretreatment sequences of response and non-response groups is the same; Hypothesis 2: the positions are independent with each other, and the pretreatment sequences of response and non-response groups have different probability distributions; Hypothesis 3: the positions are dependent, and the distribution of the pretreatment sequences of response and non-response groups is the same; Hypothesis 4: the positions are dependent, and the pretreatment sequences of response and non-response groups have different probability distributions.

We applied Bayesian Variable Partition (BVP) model and Recursive Model Selection (RMS) procedure to the pretreatment sequences of response (47 sequences) and non-response (29 sequences) samples, as described in detail in the methods section. We run the proposed method with multiple random restarts, and multiple chains will converge to different multiple local modes. So given different results, we consider all of them meaningful. We did not do any heuristic or subjective selection. Table 3 shows the results of the analysis, namely, positions which have 95% or more probability for us to infer one of our four hypotheses. As shown in Table 3, the results are not uniform from different Markov Chains. The limited sample size of our analysis may be the reason for this inconsistency. However, looking at the common positions from almost all the 20 chains still gives us a reliable idea of the mutation mechanism of positions 49, 349, and 199, 209, 242, 398 which have the highest frequencies among these 20 Markov chains. Positions 49 and 349 are statistically different in response and non-response patients and are independent of other positions. Positions 199, 209, 242, 398 are dependent and demonstrate significant difference in response and non-response patients. Position 49 is in membrane attachment region; Position 349 is in the region between V3 and V4; Positions 199 and 209 are in membrane attachment region; Position 242 is in ISDR region; Position 398 is in V3 region. These positions may have some biological influence on drug resistance to IFN and ribivirin.

**Figure 3:**
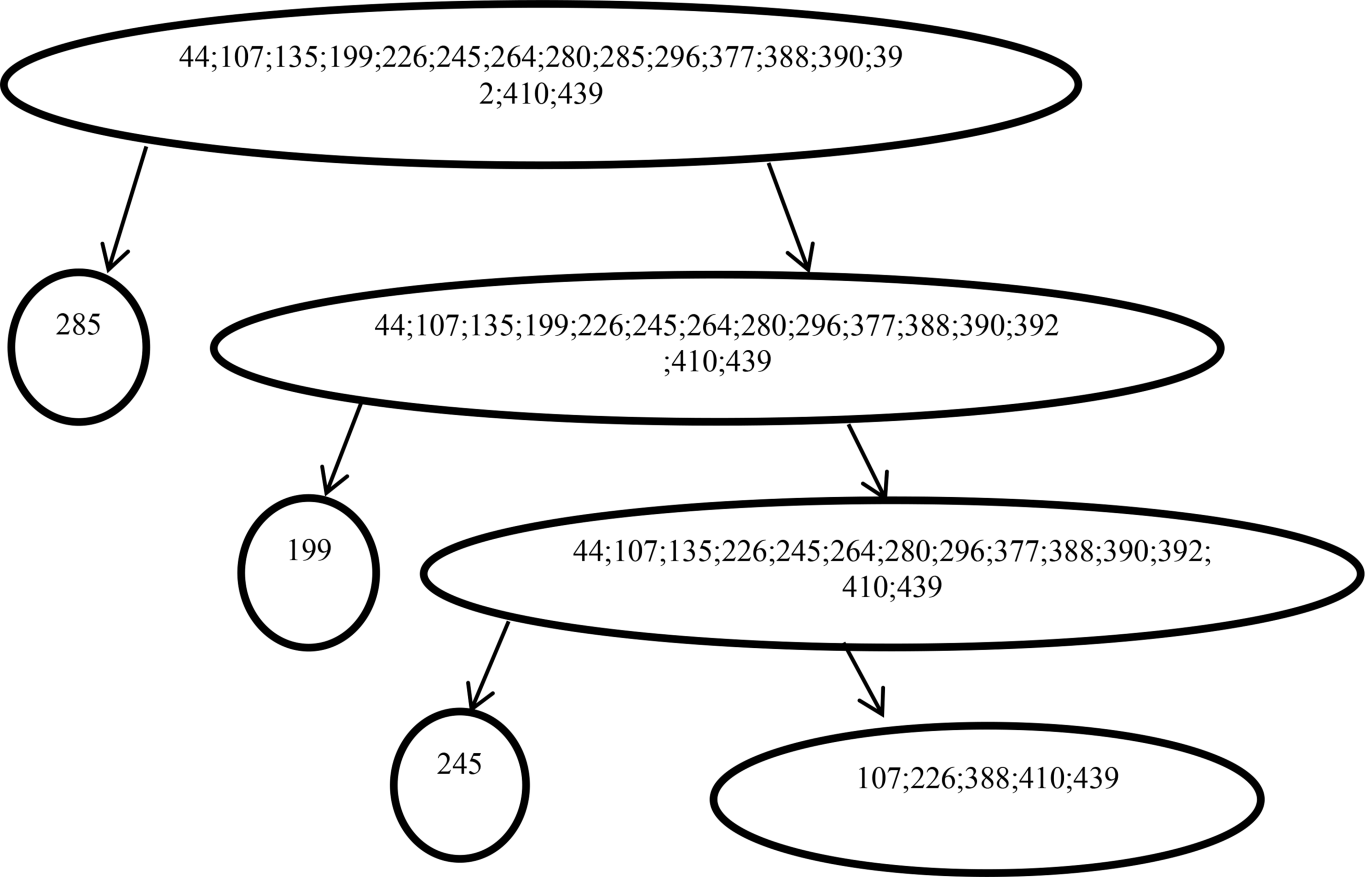
Flowchart of detected mutation positions and position combinations in the pretreatment sequence of patients who respond to the treatment.

**Figure 4:**
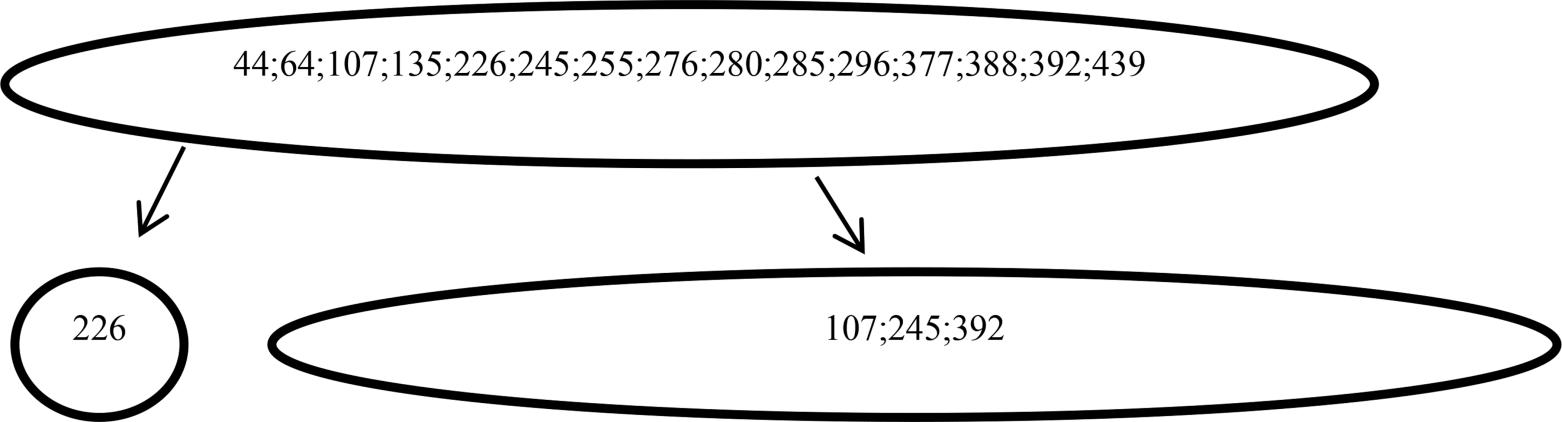
Flowchart of detected mutation positions and position combinations in the pretreatment sequence of patients who don’t respond to the treatment.

While analyzing single positions as above is helpful, a lot of positions are not mutating independently. [Figure 3] shows the interacting positions detected by BVP in response samples and [Figure 4] shows the interacting positions detected by BVP in non-response samples. Table 4 and Table 5 show the dependence structure inferred by RMS in detail, for the response and non-response group respectively. At position 285, we found that the frequency of amino acid E is 13.8% in non-response samples and 8.5% in the response samples. A more significant result was found at position 199, where the frequency of amino acid L decrease from 100% to 87.2%, from non-response samples to response samples. Similar yet less significant patterns were found at position 226, where the amino acid M decrease from 20.75 to 14.9%, from non-response samples to response samples. For dependent positions, we observed similar results, as shown jointly in Table 4 and Table 5. At positions 107, 226, 288, 410, 439, the amino acid combination EMIAE does not exist in response samples, which indicates that those positions combined may be a distinguishing factor for response and non-response patients. There are other non-existent combinations at those positions. For instance, KEIAG, TMVAG, TLIAE, are all combinations that only exist in non-response samples.

**Figure 5:**
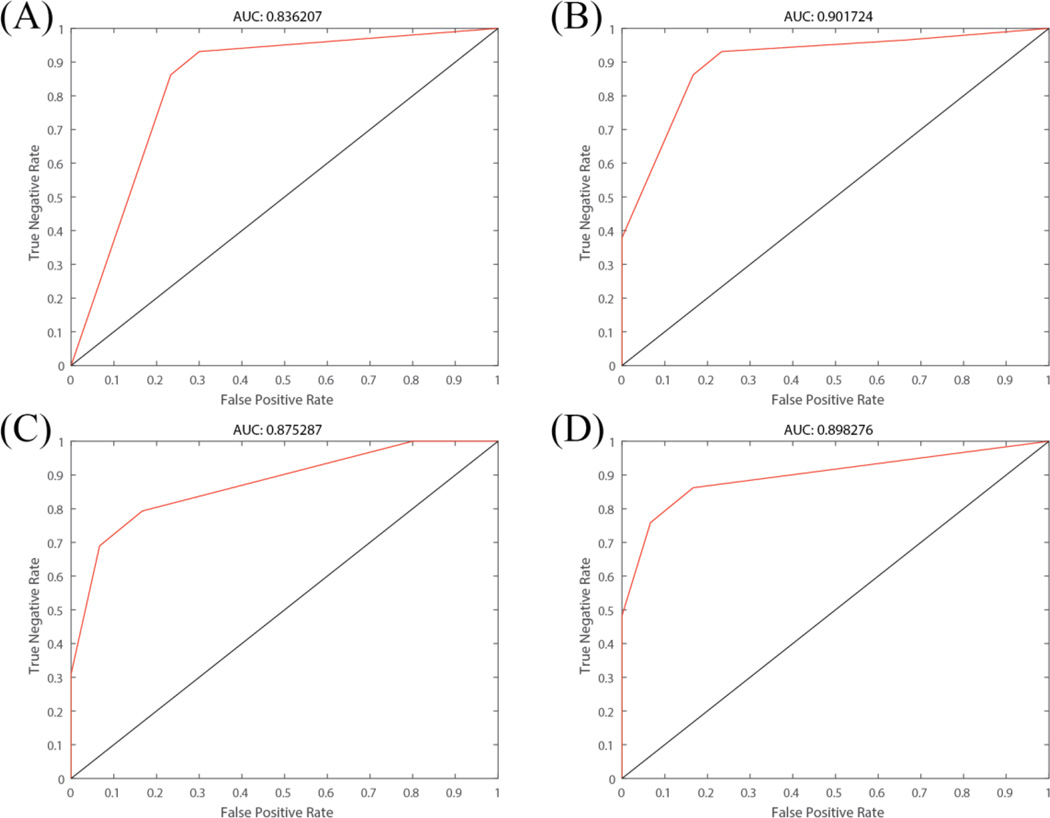
ROC curve by using SVM with selected mutations as features. (A) Mutation positions: 44, 133, 226, 269, 276, 285, 288, 296, 304, 305, 382; (B) Mutation positions: 24, 44, 107, 133, 135, 226, 304, 305, 422; (C) Mutation positions: 23, 44, 61, 64, 71, 135, 245, 269, 276, 285, 291, 382, 388, 439, 445; (D) Mutation positions: 107, 226, 388, 410, 439.

To show the prediction power of the reported mutations by our method, an SVM classifier was used to conduct a classification on the non-response and response sequences. The ROC curves and corresponding AUC values were showed in Figure 5. The SVM was implemented by libsvm^[16]^, and the hyper- parameters were tuned to be optimal by the grid search. The kernel function applied was Radial basis function, which gave best results comparing to linear, polynomial, and sigmoid kernel functions. We chose four mutation positions combinations to illustrate the prediction power of our findings: (A) 44, 133, 226, 269, 276, 285, 288, 296, 304, 305, 382 (B) 24, 44, 107, 133, 135, 226, 304, 305, 422 (C) 23, 44, 61, 64, 71, 135, 245, 269, 276, 285, 291, 382, 388, 439, 445; (D) 107, 226, 388, 410, 439. From the ROC curves and AUC values, these mutation positions demonstrate considerable discrimination and prediction power with even small samples size (47 response sequences versus 29 non-response sequences).

Single positions significant under Fisher test also reveal differences between response and non-response samples in terms of the frequency of amino acid. At position 48, the frequency of amino acid R is 100% in non-response samples, while only 70.2% in response samples. At position 81, the frequency of amino acid R is about 15% higher in response samples. These results, combined with the more reliable evidence from Table 4 and Table 5, gave us a relatively complete picture of the differences between non-response and response samples.

## Discussion

Utilizing the pioneering method proposed by Zhang et al^[14]^, which employs Bayesian statistical modeling, we were able to detect and analyze, the complex interactions of mutations of the HCV protease and reverse transcriptase.

While this analysis helps present a relatively comprehensive picture of the different pretreatment structures of non-response and response patients, it admittedly omits many other factors that possibly influence HCV virus mutations. Despite all the possibilities that may emerge, this study has not only confirmed the original findings of HCV drug resistance but also demonstrated the long-puzzled selection pattern of HCV drug treatment effects. We are positive that the method and results presented here will make a stimulation of new and more accurate ways to decipher the myths behind drug resistance of HCV and other related diseases.

## Supplementary Material

JBPR-15-RA-001.Suppl.Fig

JBPR-15-RA-001.Suppltables

## Figures and Tables

**Figure 1:**
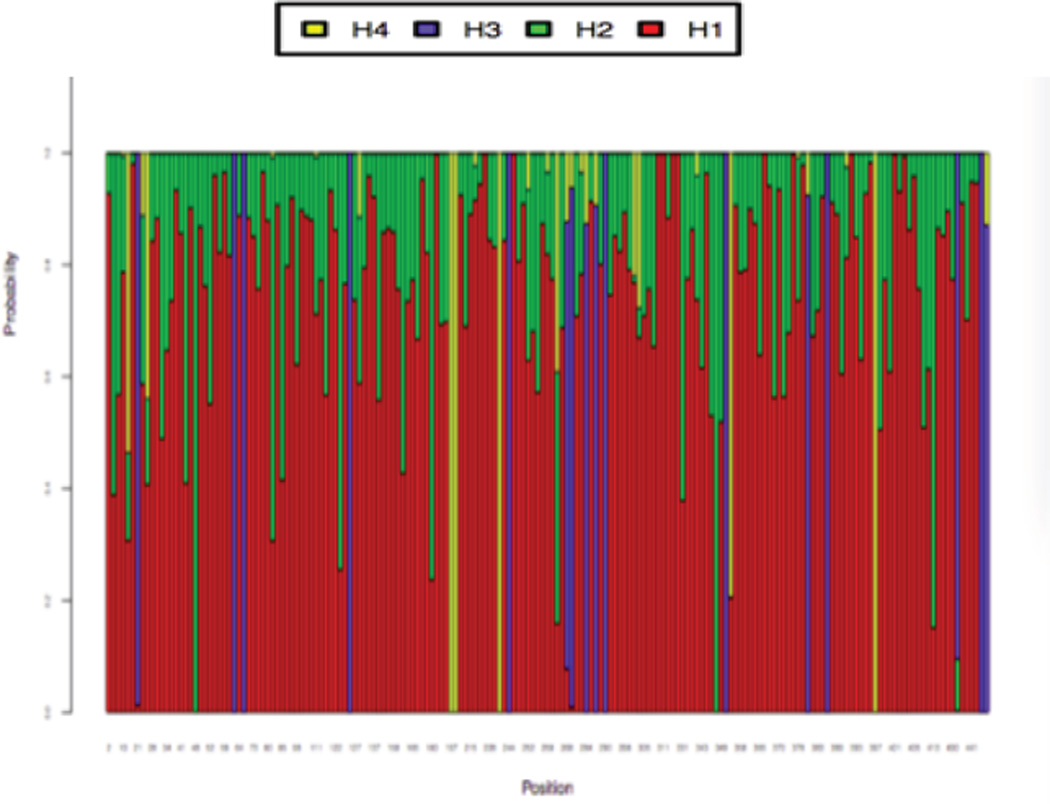
Hypotheses distribution (H1, H2, H3 and H4) in a sample Markov Chain.

**Figure 2:**
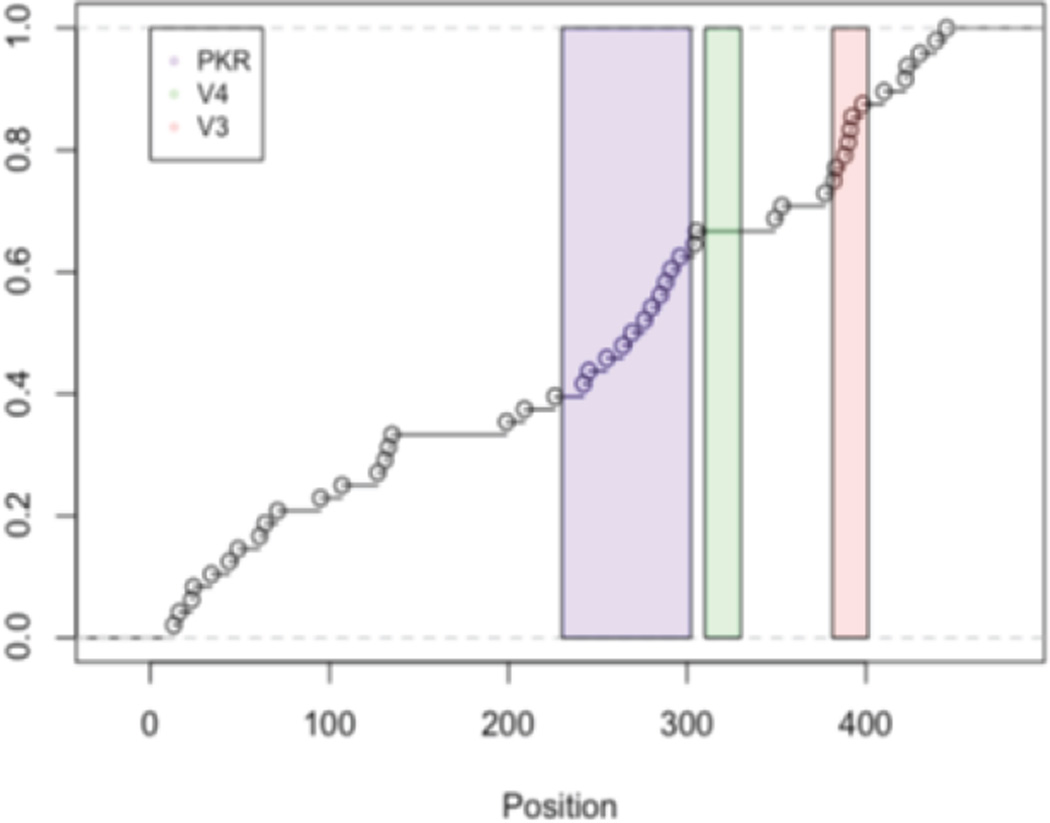
Summary for Table 3

**Table 1:** Accession numbers for 47 response sequences.

Response Sequences					
	Accession number	Accession number		Accession number	
1	AF265047	17	AM600927	33	EF407419
2	AF265111	18	AM600936	34	EF407420
3	AF265129	19	AM600919	35	EF407421
4	AF265132	20	AM600923	36	EF407422
5	AF265138	21	AM600950	37	EF407423
6	AM600953	22	AM600914	38	EF407424
7	AM600938	23	AM600930	39	EF407425
8	AM600925	24	AM600944	40	EF407411
9	AM600932	25	AM600951	41	EF407412
10	AM600929	26	AM600942	42	FJ958369
11	AM600921	27	EF407413	43	FJ958414
12	AM600955	28	EF407414	44	FJ958465
13	AM600934	29	EF407415	45	FJ958543
14	AM600946	30	EF407416	46	FJ958850
15	AM600948	31	EF407417	47	FJ958939
16	AM600940	32	EF407418		

**Table 2:** Accession numbers for 29 non-response sequences

Non-response Sequences					
	Accession Number	Accession Number		Accession Number	
1	EF407432	11	AF265105	21	EF407434
2	EF407437	12	AF265009	22	EF407435
3	EF407445	13	AF265028	23	EF407436
4	EF407427	14	EF407427	24	EF407437
5	EF407430	15	EF407428	25	EF407438
6	EF407436	16	EF407429	26	EF407439
7	AF265141	17	EF407430	27	EF407440
8	AF265135	18	EF407431	28	EF407441
9	AF265121	19	EF407432	29	EF407442
10	AF265117	20	EF407433		

**Table 3:** Positions whose posterior probabilities of H2 or H4 are larger than 0.95.

# Chain	H2 (P > 0.95)	H4 (P > 0.95)
1	49 349	23 64 71 245 269 276 288 291 382 388 445
2	49 349	13 390 391
3	49 349	34 242 377 398
4	Null	44 133 226 269 276 285 288 296 304 305 382
5	49 349	61 64 135 296 423 430
6	49 349	16 23 61 135 226 255 388
7	49 349	199 209 242 398
8	49 349	24 44 107 133 135 226 304 305 422
9	49 349	23 44 61 64 71 135 245 269 276 285 291 382 388 439 445
10	49 349	133 264 280 305 392
11	49	16 95 131 383 410 439
12	49 349	34 199 209 242 377 398
13	49 349	71 127 245 269 276 285 288 353 439 445
14	49 349	23 64 71 245 269 276 285 288 291 382 388 445
15	349	242 390 391 398
16	49 349	Null
17	49 349	34 95 135 255 377
18	49 349	199 209 242 398
19	49 349	133 269 276 285 288 304 305 382
20	49 349	199 209 242 398

**Table 4:** Detailed position interaction relations for positions for the pretreatment sequence of patients who respond to the treatment.

Positions	Amino Acids Frequency									
285	E	D	V							
Non-response	13.80%	86.20%	0.00%							
response	8.50%	89.40%	2.10%							
199	L	V								
Non-response	100.00%	0.00%								
response	87.20%	12.80%								
245	A	T	V	N	Y					
Non-response	44.80%	48.30%	6.90%	0.00%	0.00%					
response	29.80%	63.80%	2.10%	2.10%	2.10%					
107/226/388/410/439	E+M+I+A+E	K+L+I+A+E	T+V+I+A+E	T+L+I+A+G	K+E+I+A+G	T+M+V+A+G	T+L+I+A+E	T+V+I+A+G	T+E+I+V+G	T+M+I+A+G
Non-response	6.90%	6.90%	10.30%	6.90%	10.30%	6.90%	10.30%	6.90%	3.40%	3.40%
response	0.00%	2.10%	8.50%	8.50%	0.00%	0.00%	0.00%	19.10%	0.00%	0.00%
107/226/388/410/439	K+V+V+A+G	K+M+I+A+G	M+E+I+A+E	K+V+I+A+E	T+E+I+A+G	K+V+I+A+G	K+V+V+A+E	T+M+I+A+E	K+M+I+A+E	K+L+I+D+E
Non-response	6.90%	3.40%	3.40%	3.40%	3.40%	3.40%	3.40%	0.00%	0.00%	0.00%
response	0.00%	0.00%	0.00%	2.10%	14.90%	6.40%	0.00%	2.10%	6.40%	2.10%
107/226/388/410/439	S+V+I+A+E	T+M+V+A+E	T+L+T+A+E	K+V+I+G+E	T+W+T+A+D	T+M+V+S+G	T+V+I+T+E	E+M+I+A+G	K+E+A+A+E	T+E+I+A+E
Non-response	0.00%	0.00%	0.00%	0.00%	0.00%	0.00%	0.00%	0.00%	0.00%	0.00%
response	2.10%	2.10%	2.10%	2.10%	2.10%	2.10%	4.30%	2.10%	2.10%	2.10%
107/226/388/410/439	K+L+V+A+G	T+E+V+A+G								
Non-response	0.00%	0.00%								
response	2.10%	2.10%								

**Table 5:** Detailed position interaction relations for positions for the pretreatment sequence of patients who don’t respond to the treatment.

Positions	Amino Acids Frequency									
226	M	L	V	E	W					
Non-response	21.70%	11.50%	49.30%	0.70%	0.70%					
response	24.00%	18.00%	38.00%	6.00%	0.00%					
107/245/392	E+A+N	K+A+N	T+A+N	T+T+D	K+T+N	T+T+N	T+V+S	T+V+D	M+A+D	K+T+S
Non-response	6.90%	17.20%	6.90%	6.90%	10.30%	27.60%	3.40%	3.40%	3.40%	3.40%
response	2.10%	8.50%	12.80%	17.00%	8.50%	34.00%	0.00%	0.00%	0.00%	2.10%
107/245/392	T+A+D	K+A+D	T+T+V	S+A+N	T+N+N	K+V+N	K+Y+N			
Non-response	3.40%	6.90%	0.00%	0.00%	0.00%	0.00%	0.00%			
response	2.10%	2.10%	2.10%	2.10%	2.10%	2.10%	2.10%			
