# Supplementary material for: Analyzing the Effects of Pretreatment Diversity on HCV Drug Treatment Responsiveness Using Bayesian Partition methods
Source: J Bioinform Proteom Rev. Author manuscript; Available in PMC 2015 Oct 8. (PMC4597793)
Supplement: JBPR-15-RA-001.Suppl.Fig [file NIHMS700864-supplement-JBPR-15-RA-001_Suppl_Fig.docx]

## Supplementary Materials

**Figure 1:** Hypotheses distribution (H1, H2, H3 and H4) in a sample Markov Chain.

**Figure 2:** Summary for Table 1
