# Supplementary material for: Analyzing the Effects of Pretreatment Diversity on HCV Drug Treatment Responsiveness Using Bayesian Partition methods
Source: J Bioinform Proteom Rev. Author manuscript; Available in PMC 2015 Oct 8. (PMC4597793)
Supplement: JBPR-15-RA-001.Suppltables [file NIHMS700864-supplement-JBPR-15-RA-001_Suppltables.docx]

**Table 1:** Positions whose posterior probabilities of H2 or H4 are larger than 0.95

| #Chain | H2 (P>0.95) | H4 (P> 0.95) |
| --- | --- | --- |
| 1 | 49 349 | 23 64 71 245 269 276 288 291 382 388 445 |
| 2 | 49 349 | 13 390 391 |
| 3 | 49 349 | 34 242 377 398 |
| 4 | Null | 44 133 226 269 276 285 288 296 304 305 382 |
| 5 | 49 349 | 61 64 135 296 423 430 |
| 6 | 49 349 | 16 23 61 135 226 255 388 |
| 7 | 49 349 | 199 209 242 398 |
| 8 | 49 349 | 24 44 107 133 135 226 304 305 422 |
| 9 | 49 349 | 23 44 61 64 71 135 245 269 276 285 291 382 388 439 445 |
| 10 | 49 349 | 133 264 280 305 392 |
| 11 | 49 | 16 95 131 383 410 439 |
| 12 | 49 349 | 34 199 209 242 377 398 |
| 13 | 49 349 | 71 127 245 269 276 285 288 353 439 445 |
| 14 | 49 349 | 23 64 71 245 269 276 285 288 291 382 388 445 |
| 15 | 349 | 242 390 391 398 |
| 16 | 49 349 | Null |
| 17 | 49 349 | 34 95 135 255 377 |
| 18 | 49 349 | 199 209 242 398 |
| 19 | 49 349 | 133 269 276 285 288 304 305 382 |
| 20 | 49 349 | 199 209 242 398 |

**Table 2:** Detailed position interaction relations for positions for the pretreatment sequence of patients who respond to the treatment

| Positions | Amino Acids Frequency | | | | | | | | | |
| --- | --- | --- | --- | --- | --- | --- | --- | --- | --- | --- |
| 285 | E | D | V |  | | | | | | |
| Non-response | 13.80% | 86.20% | 0.00% |  |  |  |  |  |  |  |
| response | 8.50% | 89.40% | 2.10% |  |  |  |  |  |  |  |
| 199 | L | V |  | | | | | | | |
| Non-response | 100.00% | 0.00% |  |  |  |  |  |  |  |  |
| response | 87.20% | 12.80% |  |  |  |  |  |  |  |  |
| 245 | A | T | V | N | Y |  | | | | |
| Non-response | 44.80% | 48.30% | 6.90% | 0.00% | 0.00% |  |  |  |  |  |
| response | 29.80% | 63.80% | 2.10% | 2.10% | 2.10% |  |  |  |  |  |
| 107/226/388/410/439 | E+M+I+A+E | K+L+I+A+E | T+V+I+A+E | T+L+I+A+G | K+E+I+A+G | T+M+V+A+G | T+L+I+A+E | T+V+I+A+G | T+E+I+V+G | T+M+I+A+G |
| Non-response | 6.90% | 6.90% | 10.30% | 6.90% | 10.30% | 6.90% | 10.30% | 6.90% | 3.40% | 3.40% |
| response | 0.00% | 2.10% | 8.50% | 8.50% | 0.00% | 0.00% | 0.00% | 19.10% | 0.00% | 0.00% |
| 107/226/388/410/439 | K+V+V+A+G | K+M+I+A+G | M+E+I+A+E | K+V+I+A+E | T+E+I+A+G | K+V+I+A+G | K+V+V+A+E | T+M+I+A+E | K+M+I+A+E | K+L+I+D+E |
| Non-response | 6.90% | 3.40% | 3.40% | 3.40% | 3.40% | 3.40% | 3.40% | 0.00% | 0.00% | 0.00% |
| response | 0.00% | 0.00% | 0.00% | 2.10% | 14.90% | 6.40% | 0.00% | 2.10% | 6.40% | 2.10% |
| 107/226/388/410/439 | S+V+I+A+E | T+M+V+A+E | T+L+T+A+E | K+V+I+G+E | T+W+T+A+D | T+M+V+S+G | T+V+I+T+E | E+M+I+A+G | K+E+A+A+E | T+E+I+A+E |
| Non-response | 0.00% | 0.00% | 0.00% | 0.00% | 0.00% | 0.00% | 0.00% | 0.00% | 0.00% | 0.00% |
| response | 2.10% | 2.10% | 2.10% | 2.10% | 2.10% | 2.10% | 4.30% | 2.10% | 2.10% | 2.10% |
| 107/226/388/410/439 | K+L+V+A+G | T+E+V+A+G |  | | | | | | | |
| Non-response | 0.00% | 0.00% |  |  |  |  |  |  |  |  |
| response | 2.10% | 2.10% |  |  |  |  |  |  |  |  |

**Table 3:** Detailed position interaction relations for positions for the pretreatment sequence of patients who don’t respond to the treatment

| Positions | Amino Acids Frequency | | | | | | | | | |
| --- | --- | --- | --- | --- | --- | --- | --- | --- | --- | --- |
| 226 | M | L | V | E | W |  | | | | |
| Non-response | 21.70% | 11.50% | 49.30% | 0.70% | 0.70% |  |  |  |  |  |
| response | 24.00% | 18.00% | 38.00% | 6.00% | 0.00% |  |  |  |  |  |
| 107/245/392 | E+A+N | K+A+N | T+A+N | T+T+D | K+T+N | T+T+N | T+V+S | T+V+D | M+A+D | K+T+S |
| Non-response | 6.90% | 17.20% | 6.90% | 6.90% | 10.30% | 27.60% | 3.40% | 3.40% | 3.40% | 3.40% |
| response | 2.10% | 8.50% | 12.80% | 17.00% | 8.50% | 34.00% | 0.00% | 0.00% | 0.00% | 2.10% |
| 107/245/392 | T+A+D | K+A+D | T+T+V | S+A+N | T+N+N | K+V+N | K+Y+N |  | | |
| Non-response | 3.40% | 6.90% | 0.00% | 0.00% | 0.00% | 0.00% | 0.00% |  |  |  |
| response | 2.10% | 2.10% | 2.10% | 2.10% | 2.10% | 2.10% | 2.10% |  |  |  |
